# Effects of sevuparin on rosette formation and cytoadherence of *Plasmodium falciparum* infected erythrocytes

**DOI:** 10.1371/journal.pone.0172718

**Published:** 2017-03-01

**Authors:** Somporn Saiwaew, Juntima Sritabal, Nattaporn Piaraksa, Srisuda Keayarsa, Ronnatrai Ruengweerayut, Chirapong Utaisin, Patima Sila, Rangsan Niramis, Rachanee Udomsangpetch, Prakaykaew Charunwatthana, Emsri Pongponratn, Sasithon Pukrittayakamee, Anna M. Leitgeb, Mats Wahlgren, Sue J. Lee, Nicholas P. J. Day, Nicholas J. White, Arjen M. Dondorp, Kesinee Chotivanich

**Affiliations:** 1 Department of Clinical Tropical Medicine, Faculty of Tropical Medicine, Mahidol University, Bangkok, Thailand; 2 Mae Sot Hospital, Mae Sot, Tak, Thailand; 3 Mae Ramat Hospital, Mae Ramat, Tak, Thailand; 4 Queen Sirikit National Institute of Child Health, Bangkok, Thailand; 5 Center for Research and Innovation, Faculty of Medical Technology, Mahidol University, Nakhon Pathom, Thailand; 6 Department of Tropical Pathology, Faculty of Tropical Medicine, Mahidol University, Bangkok, Thailand; 7 Dilaforette AB, Stockholm, Sweden; 8 Department of Microbiology, Tumor and Cell Biology, Karolinska Institutet, Stockholm, Sweden; 9 Mahidol-Oxford Tropical Medicine Research Unit, Faculty of Tropical Medicine, Mahidol University, Bangkok, Thailand; 10 Centre for Tropical Medicine and Global Health, Nuffield Department of Medicine, University of Oxford, Oxford, United Kingdom; Liverpool School of Tropical Medicine, UNITED KINGDOM

## Abstract

In severe *falciparum* malaria cytoadherence of parasitised red blood cells (PRBCs) to vascular endothelium (causing sequestration) and to uninfected red cells (causing rosette formation) contribute to microcirculatory flow obstruction in vital organs. Heparin can reverse the underlying ligand-receptor interactions, but may increase the bleeding risks. As a heparin-derived polysaccharide, sevuparin has been designed to retain anti-adhesive properties, while the antithrombin-binding domains have been eliminated, substantially diminishing its anticoagulant activity. Sevuparin has been evaluated recently in patients with uncomplicated *falciparum* malaria, and is currently investigated in a clinical trial for sickle cell disease. The effects of sevuparin on rosette formation and cytoadherence of *Plasmodium falciparum* isolates from Thailand were investigated. Trophozoite stages of *P*. *falciparum*-infected RBCs (Pf-iRBCs) were cultured from 49 patients with malaria. Pf-iRBCs were treated with sevuparin at 37°C and assessed in rosetting and in cytoadhesion assays with human dermal microvascular endothelial cells (HDMECs) under static and flow conditions. The proportion of Pf-iRBCs forming rosettes ranged from 6.5% to 26.0% (median = 12.2%). Rosetting was dose dependently disrupted by sevuparin (50% disruption by 250 μg/mL). Overall 57% of *P*. *falciparum* isolates bound to HDMECs under static conditions; median (interquartile range) Pf-iRBC binding was 8.5 (3.0–38.0) Pf-iRBCs/1000 HDMECs. Sevuparin in concentrations ≥ 100 μg/mL inhibited cytoadherence. Sevuparin disrupts *P*. *falciparum* rosette formation in a dose dependent manner and inhibits cytoadherence to endothelial cells. The data support assessment of sevuparin as an adjunctive treatment to the standard therapy in severe *falciparum* malaria.

## Introduction

There were an estimated 198 million cases and 584,000 deaths from malaria in 2013 [1]. The majority of infections and deaths are caused by *P*. *falciparum* [2]. New effective therapies against severe malaria are needed. Binding of *P*. *falciparum*-infected red blood cells (Pf-iRBCs) to vascular endothelium (cytoadherence) [3, 4], to infected red blood cells (platelets-induced auto-agglutination) [5, 6], and to uninfected red blood cells (rosetting) [7–9] are central to the pathophysiology of severe *P*. *falciparum* malaria. The severity of clinical infection is proportional to the degree of microvascular obstruction in vital organs (e.g. brain, lungs, kidneys and liver).

Major endothelial cell receptors identified for cytoadherence include CD36 (platelet glycoprotein IV) [10], TSP (thrombospondin) [11], ICAM-1 (intercellular adhesion molecule-1 or CD54) [12] and EPCR (endothelial protein C receptor) [13]. The CD36 receptor is expressed on the vascular endothelial cells [14]. Heparan sulfate (HS) is another important receptor present on both RBC surface and endothelium, and contributes to both rosetting and cytoadherence. HS and CD36 bind to the parasite adherence ligand of *P*. *falciparum* [15–18]. This is *P*. *falciparum* erythrocyte membrane protein-1 (PfEMP-1) [19], a polypeptide antigen of 200–350 kD encoded by the *var* gene family of *P*. *falciparum* (60 *var*-genes) [20]. PfEMP-1 contains several extracellular Duffy binding like domains (DBL 1–5), and one to two cysteine-rich inter-domain regions (CIDRs) which bind to a variety of host cell receptors [21, 22]. Specific DBL domains mediate rosetting [23, 24] by variants receptors on uninfected-RBCs, including complement receptor1 (CR1) [25] and A, B blood group tri-saccharides [26]. Some rosetting variants also bind the Fc region of human IgM [27] and alpha 2-macroglobulin that facilitate rosetting and map to the c- terminal of PfEMP-1 domain [28]. Antibodies to PfEMP-1 can disrupt rosettes and protect against the sequestration of Pf-iRBCs [26, 29–31]. Heparan sulfate and heparin bind directly to the DBL1α domain of PfEMP-1 and also inhibit rosetting and cytoadherence [17]. The cytoadherence of the HBC-EC6 parasite line to human microvascular endothelial cells (HMEC-1) can be inhibited by the negatively charged, sulfated glycoconjugates [32]. Previous studies used heparin as an adjunctive treatment of severe malaria [32–37]. However use of heparin increases risk of bleeding. Sevuparin is a negatively charged polysaccharide derived from heparin through chemical depolymerization, in which the specific pentasaccharide that is involved in high-affinity binding to antithrombin III is deleted. As a result, sevuparin has no direct effect on factor Xa nor on thrombin, and the effect on activated partial thromboplastin time (aPTT) is markedly reduced in comparison with heparin and low molecular weight heparin (LMWH) [38,39], however the inhibitory effects on rosetting and cytoadherence both *in vitro* [40] and in rat and macaque monkey models of severe malaria *in vivo* [38,41] are retained. An intravenous (i.v.) injection of sevuparin and close analogs molecules blocked up to 80% of infected red blood cells from binding in the microvasculature of the rat and also released previously sequestered parasitised red blood cells into the circulation [41]. Sevuparin was recently evaluated for safety in patients with *falciparum* malaria (Leitgeb et al, personal communication). In this study, we investigated *ex-vivo* the effects of sevuparin on rosette formation and on adhesion of Pf-iRBCs to endothelial cells under static and flow conditions. The results provide a basis for assessing sevuparin as an adjunctive treatment to the standard therapy in severe *falciparum* malaria patients.

## Results

Blood samples were obtained from 53 patients aged between 18–58 years old with *Plasmodium falciparum* malaria and parasite densities >10,000 Pf-iRBCs/μL. The mean (standard deviation; SD) age of patients was 30.23 (9.11) years and the mean (SD) haematocrit was 38.1% (4.7%). The geometric mean (95% CI) parasite density was 29,518 (23,426–37,196 Pf-iRBCs/μL). 50 samples were cultivated successfully. The three isolates which could not be assessed had very low parasitaemia, grew slowly and developed gametocytes. Of 50 samples 47 (97%) developed to trophozoites and rosetting assays were performed and 49 (98%) samples from cryopreserved parasites grew and static binding assays were performed.

### Rosetting assay

**Fig 1:**
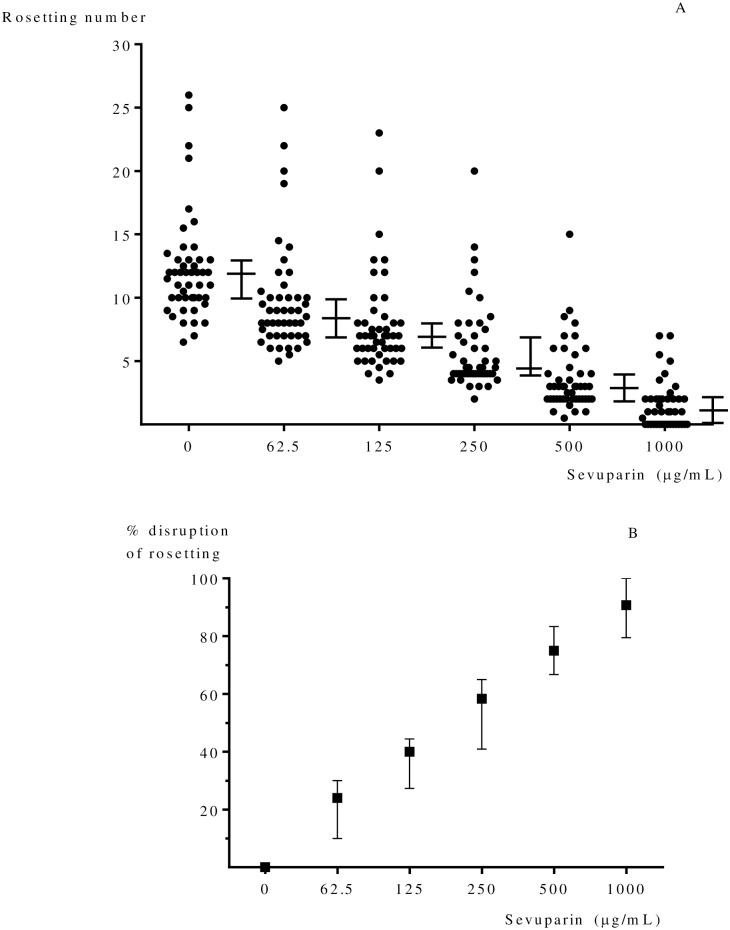
(A) Effect of sevuparin on rosetting (%) of *P*. *falciparum* (n = 47) is dose dependent. The data show the median with interquartile range of rosettes formed at each concentration of sevuparin. (B) Median (interquartile range) % disruption of rosetting at each concentration of sevuparin. Sevuparin significantly disrupted rosette formation, p < 0.001.

All *P*. *falciparum* isolates from uncomplicated malaria patients (n = 47) formed rosettes. The median (interquartile range; IQR) percentage of Pf-iRBCs forming rosettes was 12% (10.0–13.0). The median (IQR) proportion of Pf-iRBCs forming rosettes in blood group A (n = 21) was 12% (11.0–14.5), blood group B (n = 10) was 10% (8.0–12.5), blood group AB (n = 3) was 12% (8.0–13.0), and blood group O (n = 13) was 11% (9.5–13.0). There were no differences between the proportions of rosettes in the different blood groups (p = 0.205 degrees of freedom (df) = 3) and no correlation was found between numbers of rosettes and age (Spearman's (r_s_) = 0.072, p = 0.632), haematocrit (r_s_ = - 0.003, p = 0.983) and parasite density (r_s_ = 0.049, p = 0.743). Rosetting decreased with increasing sevuparin concentration *in vitro* (p < 0.001) (Fig 1A). In 16 of 42 samples (38.0%) rosetting was completely disrupted at 1000 μg/mL of sevuparin. Disruption of formed rosettes was also observed; 50% disruption of rosettes was observed at 250 μg/mL (range 125–1,000 μg/mL), (Fig 1B).

### Cytoadherence assay

**Fig 2:**
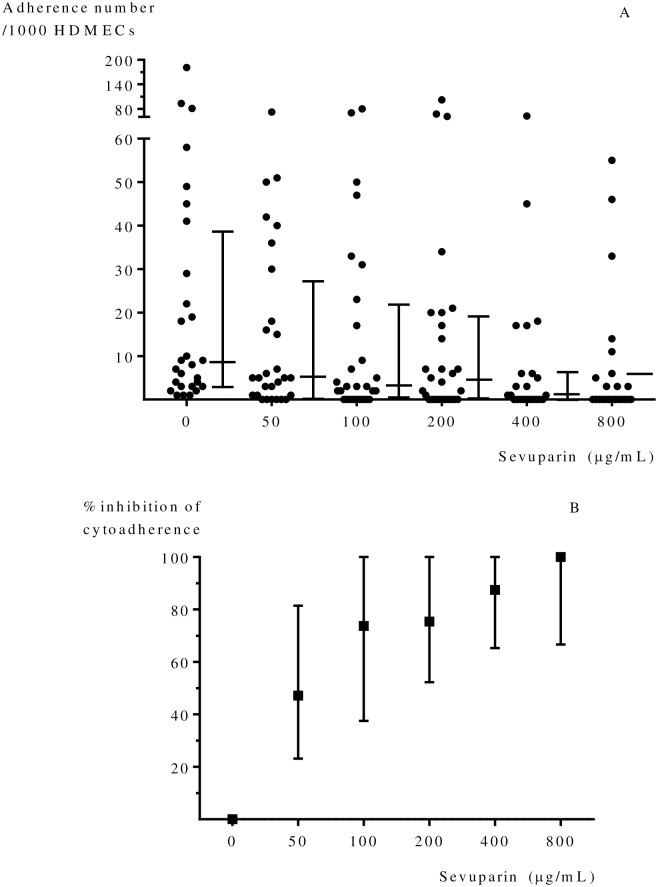
(A) The effects of sevuparin on Pf-iRBC adherence (n = 28). Median (interquartile range) adherence number of Pf-iRBCs binding per 1000 HDMECs at each concentration of sevuparin. (B) The inhibition of sevuparin on cytoadherence of *P*. *falciparum* (n = 28). Median (interquartile range) inhibition effect on cytoadhesion. The cytoadherence of patient isolates was significantly inhibited by sevuparin at concentrations ≥ 100 μg/mL (all p < 0.05).

The overall proportion of parasites from uncomplicated *P*. *falciparum* malaria which bound to human dermal microvascular endothelial cells (HDMECs) (28 of 49) was 57%. The overall median (IQR) number of Pf-iRBCs bound to HDMECs (n = 28) was 8.5 (3.0–38.0) IRBCs/1000 HDMECs, Fig 2A.

The inhibition of adhesion was calculated from the number of adherent parasites in control minus adhesion number of parasites in sample/adhesion number of parasite in control x100. There was no inhibition effect on the adhesion of parasites to endothelial cells at a sevuparin concentration of 50 μg/mL, but significant reductions were observed at higher concentrations when compared with controls (untreated or no sevuparin); 100 μg/mL (p = 0.028), 200 μg/mL (*p* = 0.040), 400 μg/mL (p < 0.001) and 800 μg/mL (p < 0.001), Fig 2B.

**Fig 3:**
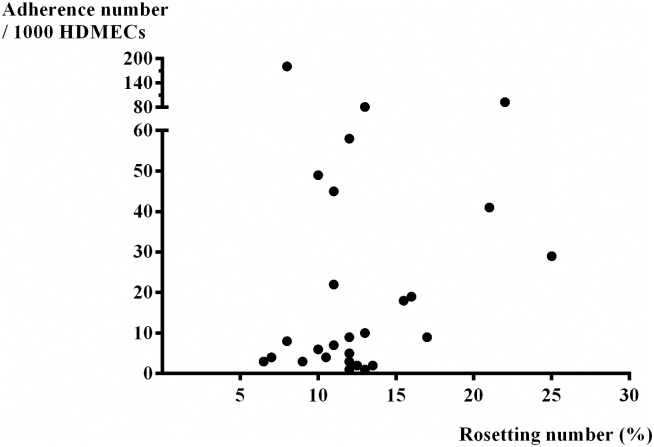
The correlation between the adherent number of Pf-iRBCs binding per 1000 HDMECs and rosetting number (%) of *P*. *falciparum* (n = 28), (r_s_ = 0.152, p = 0.439).

The median (IQR) number of Pf-iRBCs binding on HDMECs (/1000 HDMECs) in blood group A (n = 13) was 9.0 (4.0–25.5), blood group B (n = 5) was 41.0 (5.5–51.5), blood group AB (n = 3) was 81.0 (9.0–181.0) and blood group O (n = 13) was 3.0 (1.0–4.0) /1000 HDMECs). There was more adhesion of Pf-iRBCs in blood group AB when compared with the other blood groups (p = 0.020, df = 3). There was no correlation between numbers of IRBCs binding and age (r_s_ = 0.026, p = 0.896), haematocrit (r_s_ = - 0.212, p = 0.278) or parasite density (r_s_ = 0.293, p = 0.139) of patients. There was no correlation between the number of rosettes and the number of Pf-iRBCs binding to HDMECs (r_s_ = 0.152, p = 0.439), Fig 3.

### Flow based endothelial cells adhesion assay

#### Adhesion of Pf-iRBCs

**Fig 4:**
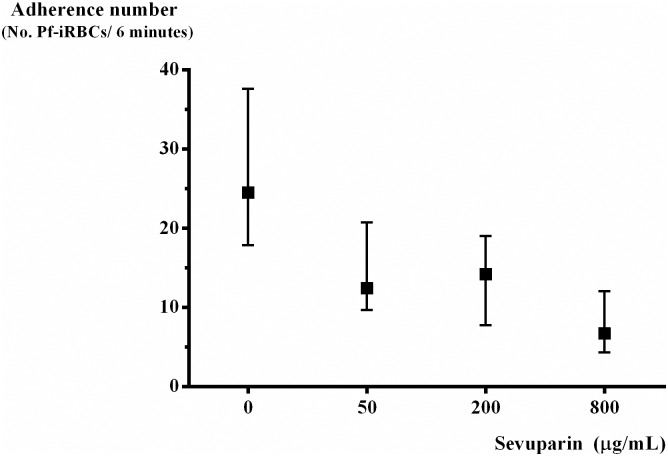
Median (interquartile range) Pf-iRBCs adhering to HDMECs under shear stress at 0.05 Pa, 0.5% parasitaemia and 1% haematocrit at different concentrations of sevuparin (n = 10).

Ten parasite isolates were treated with sevuparin at concentration 50, 200 and 800 μg/mL and assessed under shear stress at 0.05 Pa, 0.5% parasitaemia and 1% haematocrit. The median (IQR) number of Pf-iRBCs adhering to HDMECs (untreated, n = 10) was 24.5 (18.0–37.5) Pf-iRBCs/6 minutes. Following sevuparin treatment, as concentrations increased from 50, 200 to 800 μg/mL, the median (IQR) number of parasites adhering to HDMECs progressively decreased 12.4 (9.8–20.5), 14.2 (8.8–18.5) and 6.7 (4.7–10.8) Pf-iRBCs/6 minutes, respectively (Fig 4), the overall trend of median adhesion values decreased as sevuparin concentration increased p < 0.001. There was no correlation between the number of Pf-iRBCs adhering to HDMECs in relation to blood group of Pf-iRBCs (r_s_ = - 0.006, p = 0.986), age (r_s_ = 0.105, p = 0.774), haematocrit (r_s_ = 0.152, p = 0.676) or parasite density (r_s_ = - 0.100, p = 0.798).

#### Rolling of Pf-iRBCs

The median (IQR) number of Pf-iRBCs rolling on HDMECs (untreated or no sevuparin, n = 10) was 1 (1.0–2.3) Pf-iRBCs/6 minutes. The median (IQR) rolling were 1 (1.0–3.2), 1 (1.0–1.0), and 0.4 (0.0–4.0) Pf-iRBCs/6 minutes at concentrations 50 (n = 6), 200 (n = 5) and 800 (n = 3) μg/mL (Pf-iRBCs/6 minutes), respectively. The median (IQR) of Pf-iRBCs rolling was not different across the sevuparin concentrations (p = 0.504, df = 3). There was no correlation between number of Pf-iRBCs rolling on HDMECs with blood group of Pf-iRBCs (r_s_ = 0.175, p = 0.707), age (r_s_ = - 0.070, p = 0.882), hematocrit (r_s_ = 0.256, p = 0.579) or parasite density (r_s_ = - 0.374, p = 0.408). Moreover, there was a borderline association between adherent Pf-iRBC adherence and the number of rolling Pf-iRBCs (r_s_ = 0.729, p = 0.063).

#### The tethering of Pf-iRBCs

Only one isolate showed tethering adhesive phenotype at 8.7 Pf-iRBCs/6 minutes at concentration 50 μg/mL of sevuparin and 6.0 Pf-iRBCs/6 minutes at concentration 800 μg/mL of sevuparin.

## Discussion

The propensity of *P*. *falciparum*-infected red blood cells (Pf-iRBCs) to adhere to vascular endothelium and other RBCs (rosette formation) is thought to be a major factor accounting for the virulence of this parasite [42]. Both of these processes contribute to microvascular obstruction. The potentially lethal syndrome of cerebral malaria is specifically associated with cerebral sequestration [3, 43] and with increased rosetting [44], rosetting is not always associated with cerebral malaria [45, 46]. *In vivo* studies of parasite development indicate that the prognosis of severe malaria is related to the sequestered malaria parasite biomass [47]. Severely ill patients require parenteral antimalarial therapy, and any adjunctive treatment also needs to be given parenterally. From previous studies, sevuparin prevents Pf-iRBC adhesion and so has potential as an adjunct treatment of severe malaria [40]. Sevuparin both blocked and reversed sequestration of Pf-iRBC *in vivo* in rats and monkeys [38]. Sevuparin also inhibited merozoite invasion *in vitro* [38].

We investigated the effect of sevuparin on rosette formation and cytoadherence of *P*. *falciparum* isolates from uncomplicated malaria patients. In the current study, sevuparin concentrations ranged from 62.5–1000 μg/mL in the rosetting experiments and from 50–800 μg/mL in the HDMEC cytoadherence experiments, which were based on *in vitro* concentration ranges published in the literature [17, 24, 40,48,49]. Studies in healthy volunteers show that sevuparin in an i.v. dose of 5.3 mg/kg results in mean (SD) maximal plasma concentrations (Cmax) of 116 (19.3) μg/mL. Dosing in clinical studies in patients with falciparum malaria has ranged from 1.5 to 6 mg/kg per dose (unpublished). The concentration range in our in-vitro experiments range from 50–1000 μg/mL and e.g. sevuparin concentrations of 100 μg/mL showed clear inhibition of cytoadhesion, which is within the concentration range obtainable in patients (Leitgeb et al, personal communication). Sevuparin significantly disrupted the number of rosettes formed in a dose dependent manner. Rosette and cytoadherence disruption by heparin, heparan sulfate (HS) and sevuparin, and the binding of these glycosaminoglycans (GAGs) to the DBL1α domain of PfEMP-1 are well described [16–18, 24, 38, 48]. Some of these sulphated polysaccharides not only disrupt rosetting, but also inhibit adhesion of Pf-iRBCs to the endothelial and placental receptors such as chondroitin sulphate A (CSA) and CD36 [50, 51]. However, the anti-coagulant effects of heparin are an important drawback for its clinical use in severe malaria, since this is always accompanied by significant thrombocytopenia, and has been associated with an increased bleeding risk [36, 37, 52].

From previous studies, Leitgeb and colleague [40] reported > 50% rosetting disruption in 53% (25/47) of tested samples and ≥ 15% disruption in 77% (36/47) of fresh clinical isolates in Cameroon at a sevuparin concentration of 100 μg/mL. Carlson and others [49] found that 30% (16/54) of parasite samples collected in The Gambia showed ≥ 50% rosetting disruption with heparin, and 50% (27/54) of the samples showed ≥ 15% disruption at a concentration of 650 μg/mL. In this study in Thailand we found ≥ 15% rosette disruption of isolates from uncomplicated malaria (43/47) at a concentration 125 μg/mL. Complete disruption was observed in 38% (16/42) of samples at 1000 μg/mL of sevuparin. This finding is consistent with previous reports that some isolates are insensitive to heparin, heparin derivatives, and other rosette-disrupting sulfated glycoconjugates, even at very high concentrations [38, 49, 53].

CD36 binding is a property of almost all *P*. *falciparum* isolates obtained freshly from malaria patients. In the current in vitro study, 43% of fresh parasite isolates did not cytoadhere to our HDMEC cell culture expressing CD36 and the level of binding was low, varying between 1–181 infected red blood cells/1000 HDMECs. This is comparable with Udomsangpetch et al, 1996 (over 60% of isolates did not adhere to CD36) [54]. Part of the explanation could be that some VAR genes code for PfEMP1 variants that do not bind to CD36, estimated as 10 out of the 60 different *P*. *falciparum* VAR genes [21]. Studies in Africa have found no difference in CD36-binding ability between parasite isolates from severe and uncomplicated malaria patients [45, 55]. More than 90% of clinical parasite isolates tested adhere to CD36, whereas, 10% adhere to ICAM-1. For isolates that adhere to both molecules, the degree of adhesion to CD36 is at least 10-fold higher than adhesion to ICAM-1 [54]. In Thailand, two small studies showed a significant positive correlation between CD36-binding and presentation with severe malaria [56, 57]. A single human genetics study on CD36 polymorphisms in South East Asia (SEA) reported that CD36 deficiency was protective against cerebral malaria [58]. Not all studies support the importance of CD36 in mediating severe disease; a study by Rogerson et al showed preferential binding to ICAM-1 rather than CD36 in parasite isolates from paediatric severe malaria [55].

Binding of PfEMP-1 to endothelium is promiscuous and mediated by a range of membrane receptors, including Endothelial Protein-C Receptor (EPCR), thrombospondin, intercellular adhesion molecule-1 (ICAM-1), heparan sulphate (HS) and CD36. There is a wide range of literature on the importance of different receptors in mediating severe disease [21, 59]. More recently, binding of the N-terminal cysteine-rich inter domain region (CIDRα1) of PfEMP-1 to Endothelial Protein-C Receptor (EPCR) has been associated with severe malaria [13]. So, although the central role of CD36 in parasite cytoadherence is not disputed, additional receptors might play an important role in parasite sequestration in the microcirculation. Detailed studies of PfEMP-1 binding have revealed that the level of binding to its receptors is dependent on PfEMP-1 domain architectures, in particular of the Duffy Binding-like (DBL) and CIDR domains (reviewed in Smith et al.). Most PfEMP-1 (84%) will bind to CD36, and mainly carry the DBLα0-CIDRα2–6 domains. How heparin sulfate-like substance including sevuparin can inhibit or reverse these binding properties, remains to be fully elucidated. Heparan sulfate, and HS-like GAG, expressed on uninfected RBCs are receptors for rosetting [17, 60, 61]. Sevuparin might act as a decoy receptor for CD36, thus causing binding to the drug rather than to the endothelial receptor. Heparan sulfate, and thus likely also sevuparin, binds to the DBL1α domain of PfEMP-1, which could in addition directly affect both rosetting and cytoadherence. [17,18]. It could also affect cytoadherence to parasite PfEMP-1—DBL1α receptors beyond CD36. However, a recent study using co-crystallization showed that the CIDR-domain of PfEMP-1 interacts closely with the hydrophobic pocket in CD36 [62], questioning the central role of the DBL1α domain. The interference of HDMEC binding observed in our study could thus also be mediated by the effect of sevuparin on binding to other endothelial receptors.

Pf-iRBCs tethered, rolled, and adhered on human microvascular endothelial cells. Under shear stress conditions, sevuparin significantly inhibited cytoadherence to HDMECs. A previous study reported that Pf-iRBCs interaction was synergistic with multiple adhesion molecules on vascular endothelial cells whereas adhesion of Pf-iRBCs to HDMECs was largely CD36 dependent, while the rolling could be mediated by any of the adhesion molecules [63]. Pf-iRBCs adherence to HDMECs was significantly inhibited by sevuparin under flow conditions. Inhibition of adhesion was most effective with higher sevuparin doses. Previous work has shown that sevuparin reverses sequestration of IRBCs in an animal model [38]. Our current study clearly showed inhibition of binding, but did not address reversal of cytoadherence. De-sequestration with sevuparin has been described in a monkey model [38]. Mimicking this in the in-vitro model will require additional studies. In conclusion, sevuparin disrupts *P*. *falciparum* rosette formation and cytoadherence to HDMECs in a dose dependent manner and therefore might be a candidate for adjunctive therapy in severe *falciparum* malaria.

## Materials and methods

### Ethics statement

Blood samples were obtained from patients with uncomplicated *P*. *falciparum* infections admitted to Mae Sot and Mae Ramat Hospital, Tak province, Thailand. Written informed consent was obtained from all patients before blood sampling. Ethical approval was obtained from the Ethics Committee of Faculty of Tropical Medicine, Mahidol University, Bangkok, Thailand and the Ministry of Public Health of the Royal Government of Thailand, and from the Oxford Tropical Research Ethics Committee (OxTREC) of Oxford University, Oxford, UK as part of a larger clinical study (TSM02; registration number NCT01442168, Leitgeb et al, personal communication). Human dermal microvascular endothelial cells (HDMECs) were harvested from discarded neonatal human foreskins. The protocol was approved by the Ethics Committee of the Faculty of Tropical Medicine, Mahidol University and Queen Sirikit National Institute of Child Health, Bangkok. Thailand.

### Sample collection

Adult male and female (18–65 years old) presenting with acute uncomplicated *P*. *falciparum* malaria were considered for enrollment. Uncomplicated malaria defined as febrile disease with a positive peripheral blood malaria slide. Patients with any sign of severe malaria, determined on the basis of World Health Organization criteria [64] were not eligible.

Five millilitres of citrate anticoagulated blood samples were collected prior to the start of antimalarial drug treatment for microscopy malaria parasite identification in thick and thin blood smears and blood group assessment. Samples were then centrifuged at 2,500 rpm at 4°C for 5 minutes and plasma was stored at -80°C. The packed red blood cells were suspended and washed three times with RPMI-1640. Then Pf-iRBCs were cultured and the parasites cryopreserved. All experiments were performed during the first cycle of *in vitro* parasite growth. For the rosetting assay, all assays were done on fresh samples and 47 of 50 samples could be evaluated. Out of these, 28 cryopreserved isolates showed cytoadherence in the static assay and were further assessed for sevuparin. Out of these 28, 10 strains with most prominent binding were selected for the flow based endothelial cells adhesion assays.

### Parasites culture

*P*. *falciparum* parasites were grown in tissue culture flasks (Falcon^®^ Becton Dickinson, USA) in complete malaria culture medium (MCM) containing RPMI-1640 medium (Sigma, USA) supplemented with 25 mM HEPES (Sigma, USA), 2 g/L NaHCO_3_ (Sigma, USA), 4.5 g/L D-glucose (Sigma, USA), 0.1 mM hypoxanthine (Sigma, USA), 40 mg/L gentamicin (Gibco, USA) and 0.5% Albumax ^®^ (Gibco, USA). Parasite cultures were incubated at 37°C with 5% CO_2_. The culture medium was changed daily. Thin blood smears were prepared, fixed with absolute methanol (Merck, Germany), and stained with Field’s stain (BDH, UK). Pf-iRBCs were harvested for the experiment when the parasites had developed to the trophozoite stage (34–36) hours, 80% synchronization. Staging was done by light microscopy of 100 Pf-IRBCs, using published criteria for staging [65]. Synchronization was considered satisfactory if the number of mid-trophozoite stage-IRBC was more than 80% of the total Pf-IRBCs.

### Endothelial cell isolation and culture

Human dermal microvascular endothelial cells (HDMECs) were harvested from discarded neonatal human foreskins as described previously [63]. In brief, foreskins were dissected into 2 to 3 square millimetre (mm^2^) segments, and digested at 4°C for 16 hours in supplemented M199 containing 0.5 mg/mL collagenase type 1A (Boerhringer Mannheim Biochemicals, USA). Microvessels were released from digested tissue segments by compressing the tissue segments gently with a spatula. To remove large debris, vessel preparations were passed through a 100 mm nylon mesh (Becton Dickinson, USA). The cells were collected by centrifugation at 2000 rpm for 10 minutes, and the cell pellets were resuspended in endothelial basal medium (EBM-2) with supplements supplied by the manufacturer (Clonetics, USA), and then seeded in gelatin-coated tissue culture dishes or on cover slips. Finally, The HDMECs culture dishes were incubated at 37°C, 5% CO_2_. The experiments used HDMECs from passage 3 to 6 obtained from 5 different foreskin donors. HDMECs cultures were assessed for the endothelial marker CD31 and the parasite cytoadhesion receptor CD36 by an indirect immunofluorescence assay using FITC-mouse antihuman CD31 (BD Pharmingen, USA; No. 555445) and PE-mouse antihuman CD36 (BD Pharmingen, USA; No. 555455) (S1 Fig). 5 different HDMEC cell lines were used, which could differ in their adhesion properties. However, all 5 lines were selected and tested with reference CD36-positive binding parasite strains (Panned A4 and TM267 on HDMECs). There are not significant differences in the numbers of cytoadherence. (p = 0.239, for A4 and p = 0.974, for TM267).

### Preparation of sevuparin

Sevuparin was provided by Dilaforette AB, Sweden. Sevuparin is a compound derived from heparin. Sevuparin was diluted in RPMI-1640 to 2 mg/mL as stock solution and kept at 4°C until used. Sevuparin was diluted 2-fold for the experiments (range 62.5–1000 μg/mL).

### Rosetting assay

Trophozoite stages of *P*. *falciparum* (0.5% parasitaemia and 1% haematocrit) were treated with sevuparin (62.5 μg/mL—1000 μg/mL) at 37°C for 30 minutes. The cell suspension alone was used as control (i.e., 0 μg/mL). Rosette formation was then examined under light microscopy. The number of rosettes was counted per one hundred infected RBC under high magnification (100X objective lens). A rosette was scored if two or more uninfected red blood cells were bound to a single infected red blood cell. All samples were done in duplicate.

### Static endothelial cell adhesion assay

HDMECs at 80% confluence on 13–15 mm Thermanox coverslips (Nunc) were used. Trophozoite stages of *P*. *falciparum* (0.5% parasitaemia and 1% haematocrit) were treated with sevuparin (50 μg/mL—800 μg/mL for 1 hour before removing the drug, then cell suspensions were applied to each coverslip and incubated at 37°C for 1 hour with gentle rotation every 15 minutes. Unbound cells were removed by washing three times. After fixing with 0.5% glutaraldehyde and staining the cells with Giemsa’s stain, the coverslips were mounted using DPX mounting medium. The number of infected red blood cells binding to 1000 HDMECs was counted. Untreated parasites at the same stage of development were used as controls [32, 63, 66].

### Flow based endothelial cell adhesion assay

Five to one hundred thousand HDMECs in 1 ml of supplemented medium were plated on 35mm glass coverslips (Deckglasser, Germany) in 6 well plates. The cells were allowed to adhere for 2 h at 37°C, after which 3 ml of supplemented medium were added to each well. After 40 to 48 h of culture, confluent monolayers were rinsed in HBSS (Life Technologies) and used without delay in the laminar flow studies. In the adhesion assys, trophozoite stage *P*. *falciparum* (at 0.5% parasitaemia and 1% haematocrit) were preincubated with sevuparin at 50, 200 and 800 μg/mL of seveuparin for 1 hour, after which the drug was removed by washing 1 time by centrifucation at 2500 rpm, for 5 minutes and resuspension in RPMI-1640 to an haematocrit of 1%, after which the Pf-iRBCs suspension was used immediately.

The method was modified from that previously reported [63, 66, 67]. Briefly, monolayers of HDMECs were assembled in a parallel plate flow chamber of 220 μm gap thickness in which a uniform wall shear stress was generated. The flow chamber was mounted on the stage of inverted phase contrast microscope that is kept 37°C and IRBC interactions with monolayers, observed under 200 x-magnifications. All assays were performed at a wall shear stress of 0.05 Pa. This sheer stress (ɩ) was calculated from the flow rate (Q), the medium viscosity (ɳ), and the chamber diameters (w, width, and h, height): ɩ = 6. ɳ .Q/w.h^2^. A recording video was obtained of Pf-iRBCs binding over 6 separate fields; 6 minutes/field. The Pf-iRBCs suspension was passed over the endothelial cells coated cover glass slip for 6 minutes. Cell free medium was then flushed through the chamber at the same shear stress for 2 minutes to wash away non-adherent cells. Stationary adherent parasites were counted in at least six fields along the slide (both rolling and static) and the results were expressed as the number of adherent Pf-iRBCs/6 minute [63, 65, 66]. Tethering referred to initial contact between Pf-iRBCs and the endothelial monolayer. A rolling Pf-iRBC was defined as one that displayed a typical end-on-end rolling motion at a velocity of less than 3 mm and expressed as the number of rolling Pf-iRBCs/6 minutes. The Pf-iRBC was considered adherent if it remained stationary for more than 3 seconds. The results were expressed as the number of adherent Pf-iRBCs/6 minutes.

### Statistical analysis

All statistical analyses were performed using SPSS 18 for Windows. The data were tested for distributional assumptions by the Kolmogorov-Smirnov test. The Mann-Whitney *U-* test was used for non-normally distributed data to compare different levels of drug concentration against the controls. Comparisons across more than two groups were made using the Kruskal-Wallis test. Associations were assessed using the Spearman's rank correlation coefficient.

## Supporting information

S1 Fig Immunofluorescence analysis of endothelial cells markers on HDMECs. The figures show the expression of CD36 and CD31 on HDMECs monolayer. Magnification:200X.
(TIF)
