# Supplementary figures and images for: Effects of sevuparin on rosette formation and cytoadherence of Plasmodium falciparum infected erythrocytes
Source: PLoS One. 2017 Mar 1;12(3):e0172718. doi: 10.1371/journal.pone.0172718 (PMC5332063; doi:10.1371/journal.pone.0172718)

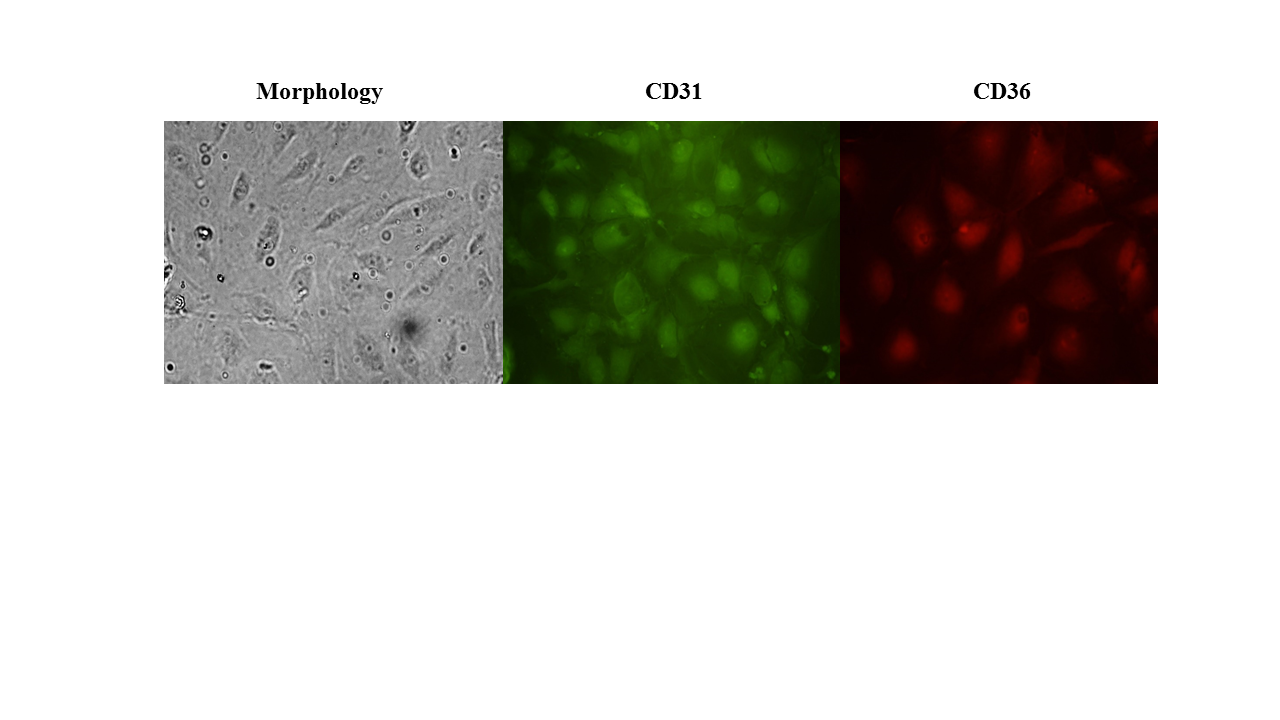

Supplement: S1 Fig — The figures show the expression of CD36 and CD31 on HDMECs monolayer. Magnification:200X. (TIF) [file pone.0172718.s001.tif]
